# High glucose induces and activates Toll-like receptor 4 in endothelial cells of diabetic retinopathy

**DOI:** 10.1186/s13098-015-0086-4

**Published:** 2015-10-13

**Authors:** Lu Wang, Jing Wang, Jiazhu Fang, Hongyan Zhou, Xialin Liu, Shao Bo Su

**Affiliations:** State Key Laboratory of Ophthalmology, Zhongshan Ophthalmic Center, Sun Yat-sen University, Guangzhou, 510060 China; Guangdong Province Hospital of Traditional Chinese Medicine, Guangzhou, 510120 China

**Keywords:** Toll-like receptor 4, Diabetic retinopathy, Endothelium, Inflammation, Angiogenesis

## Abstract

**Background:**

Hyperglycemia-induced inflammation causes the dysfunction of blood vessels, and Toll-like receptor 4 (TLR4) plays a key role in inflammation-induced angiogenesis. However, the impact of TLR4 on the pathogenesis of diabetic retinopathy (DR) is poorly understood. In this study, we examined the expression of TLR4 in retinal vascular endothelial cells of patients with DR and diabetic mice, and explored the role of TLR4 in mediating inflammatory responses by human microvascular endothelial cells (HMEC-1) under high-glucose condition.

**Methods:**

The expression of TLR4 in retinal vascular endothelial cells of patients with proliferative diabetic retinopathy and diabetic mice induced by streptozotocin was examined using immunofluorescence. HMEC-1 cells were cultured and the expression of TLR4, MyD88 and Interleukin-1β (IL-1β) was examined under high-glucose condition. Endothelial cells with TLR4 silencing and antagonist of TLR4 as well as endothelial cells from TLR4 deficient mice were used to study the effect of activated TLR4 on inflammation induced by high-glucose treatment.

**Results:**

We observed that TLR4 was detected in CD31-labled human retinal vascular endothelia and its expression was markedly increased in fibrovascular membranes from DR patients and in retinal vascular endothelial cells of diabetic mice. The expression of TLR4, MyD88 and IL-1β was enhanced by high glucose in cultured HMEC-1 and the expression of TLR4 and IL-1β was inhibited by TLR4 siRNA knock-down and TLR4 antagonist. The expression of IL-1β by endothelial cells from TLR4 deficient mice under high glucose condition was decreased.

**Conclusions:**

Our results revealed that hyperglycemia induced overexpression and activation of TLR4 in endothelial cells. This effect may lead to inflammatory responses contribute to the pathogenesis of diabetic retinopathy.

**Electronic supplementary material:**

The online version of this article (doi:10.1186/s13098-015-0086-4) contains supplementary material, which is available to authorized users.

## Background

Diabetic retinopathy (DR) is a common microvascular complication of diabetes. It remains the major cause of blindness in developed countries. Multifactors have been shown to contribute to the initiation and progression of the disease in which inflammation is pivotal. Inflammation confers an increased risk for microvascular and macrovascular complications of diabetes. The proinflammatory phenotype in DR is characterized by elevated retinal cytokines, chemokines and adhesion molecules [1, 2].

Endothelial cells are key participants in retinal ischemic vasculopathies and their dysfunction is the initial event of microvascular disorder in DR [3]. Chronic inflammatory responses in retinal endothelia cause the production of inflammatory mediators, leading to increased vascular permeability, apoptosis of endothelial cells and neovascularization [3]. Hyperglycemia, one of the major risk factors for DR, could trigger inflammation and leads to vascular complications. Upon exposure to high glucose, endothelial cells show a series of inflammatory responses, such as reduced NO production [4], but enhanced NF-κB activation [5], inflammatory gene expression [6] and leukocyte recruitment [7] by upregulated production of chemoattractants [8]. However, how hyperglycemia induces inflammation to contribute to DR remains unknown.

Toll-like receptors (TLRs) are pattern recognition receptors and recognize conserved pathogen-associated molecular patterns (PAMPs) and non-microbe molecules including endogenous ligands released during tissue damage and inflammation [9]. TLR activation triggers a signaling cascade leading to cytokine production and initiation of an adaptive immune response. TLR expression is increased in a plethora of inflammatory disorders, including diabetes [10]. Among TLRs, TLR4 recognizes lipopolysaccharide (LPS) from Gram-negative bacteria and endogenous ligands such as high-mobility group box 1 (HMGB1) and is expressed in multiple cells, such as endothelial cells and monocytes [11]. In retina, it has been reported that TLR4 was expressed in the diverse retinal cells, including retinal pigment epithelium, photoreceptors, astrocytes, microglia and retinal vascular endothelial cells [12].

TLR4 activates both the myeloid differentiation factor 88 (MyD88)-dependent pathway, which induces inflammatory cytokines, and TRIF-dependent pathway responsible for the induction of type I interferon. Several studies have shown that the expression of TLR4 is increased in atherosclerotic plaques of animal models of atherosclerosis. The lesion size, lipid content, and macrophage infiltration in atherosclerotic plaque were reduced in TLR4 knockout mice [13]. TLR4-mediated pathway also promotes dysfunction of mesangial cells that resulted in occluding of glomerular capillaries in Diabetic nephropathy [14]. Recent results suggest an association between *A*sp299*Gly* polymorphism in TLR4 gene and early onset of DR in DM2 patients. Toll-like receptor 4 polymorphisms was associated with a higher prevalence of retinopathy [15, 16]. Hyperglycemia and free fatty acids (FFAs) synergistically promoted oxidative stress to aggravated the dysfunction of endothelia and insulin resistance, which are mediated by activated TLR4 [17, 18]. Additionally, the expression of downstream factors of TLR4 including MyD88 and NF-κB, was significantly increased in cells exposed to high glucose [17, 19]. This may lead to the secretion of inflammatory cytokines including IL-1β [20]. Studies showed that the level of IL-1β was significantly elevated in vitreous fluid of patients with proliferative diabetic retinopathy and in the retina fluid of DR rats [21]. Enhanced expression of TLR4 has been shown in monocytes of diabetic patients with microvascular complications [22]. Furthermore, the studies showed the involvement of TLR4 in other human diabetic complications like nephropathy, wound healing impairment. Lin et al. reported that Toll-like receptor 4 promoted tubular inflammation in diabetic nephropathy [23]. Down-regulation of TLR4 was shown in the impairment of wound healing in T2DM patients [24, 25]. TLR4 played an important role in the initiation and progression of cardiovascular pathologies [26]. Our previous study showed that TLR4 plays a critical role in inflammation-induced angiogenesis by promoting endothelial cell sprouting, proliferation and chemotaxis in a mouse model of alkali-induced corneal neovascularization [27]. We also found that TLR4 deficiency could protect mice from angiogenesis in oxygen-induced retinopathy model [28, 29].

Accumulating evidence implicated that TLR4-mediated inflammation was involved in diabetic vascular complication. However, the role of TLR4 in mediating the pathogenesis of DR by endothelial cells remains to be elucidated. In this study, we examined the role of TLR4-dependent pathway in high glucose-induced inflammatory responses in retinal vascular endothelial cells. We demonstrate that high glucose challenge enhances the expression of TLR4 and the secretion of inflammatory factors by human endothelial cells. These results suggest an important role of hyperglycemia-induced expression and activation of TLR4 in diabetic retinopathy.

## Methods

### Patients and tissue samples

Five patients diagnosed as PDR were recruited from Zhongshan Ophthalmic Centre, Sun Yat-sen University, Guangzhou, China. Five normal human eye balls were from the Eye Bank of Zhongshan Ophthalmic Centre (Table 1). PDR patients underwent a series of ophthalmic examination, including the history of previous ocular treatments, slit-lamp biomicroscopy, gonioscopy ophthalmoscopy, fluorescein angiography, and fundus color photography. The severity of diabetic retinopathy was assessed based on the standardized fundus color photographs and on the fluorescein angiograms. The medical examination included fasting plasma concentrations of blood glucose and glycosylated hemoglobin. All patients received vitrectomy because of vitreous hemorrhage. Fibrovascular membranes from PDR patients were obtained during the surgery. The size are about 2.0 mm × 2.0 mm × 0.5 mm. After immersed in PBS for 10 s three times, the tissues were embedded in OCT for section. This research was carried out in accordance with the principles of the Declaration of Helsinki as revised in 2000. Institutional Ethics Committee approval and informed consent from all patients were obtained.

**Table 1:** Clinical characteristics for individual proliferative retinal membranes and human retina

	Age (years)	Sex	Diagnosis	Duration of diabetes (years)
Patient no.				
1	67	M	PDR epiretinal membrane	10
2	48	F	PDR epiretinal membrane	16
3	69	M	PDR epiretinal membrane	8
4	63	M	PDR epiretinal membrane	20
5	68	F	PDR epiretinal membrane	5
Control no.				
1	52	M	Normal	n/a
2	58	F	Normal	n/a
3	51	M	Normal	n/a
4	60	M	Normal	n/a
5	61	M	Normal	n/a

n/a indicates that there are no duration data for the control subjects

### Animal studies

C57BL/6 mice were purchased from Experimental Animal Centre of Sun Yat-sen University of Medical Science (Guangzhou, China). Care, use and the treatment of all animals were in strict agreement with the guidelines of the Association for Research in Vision and Ophthalmology Statement for the Use of Animals in Ophthalmic and Visual Research and approved by the institutional animal care and use committees in Zhongshan ophthalmic centre. To induce diabetes, 6-week-old C57BL/6 mice were given five consecutive intraperitoneal injections of streptozotocin (STZ; 60 mg/kg body wt/day) (Sigma-Aldrich) or vehicle as control. Six and eight weeks after injection, mice were humanely killed and eyes were embedded in OCT for section.

### Immunofluorescence

Dual-color immunofluorescence staining was performed on frozen sections of the fibrovascular membranes and mouse eye balls with a mouse anti-human or anti-mouse TLR4 monoclonal antibody (1:100 dilution; Santa Cruz Biotechnology, California, USA; 1:100 dilution; Abcam, Cambridge, MA, USA) and with rabbit anti-human/mouse CD31 polyclonal IgG (1:100 dilution; Biosynthesis Biotechnology Co, Ltd, Beijing, China). The samples were then incubated with secondary goat anti-rabbit IgG antibody Alexa Fluor 555 and a goat anti-mouse IgG antibody Alexa Fluor 488 (1:500 dilution, Cell Signaling Technology, Boston, MA, USA) for 1 h at 37 °C in the dark, followed by a 5-min incubation with Hoechst 33258 (1:1000 dilution; Sigma-Aldrich, St. Louis, Missouri, USA). Human retinal sections were examined at 400× with fluorescence microscope (Axioskop; Carl Zeiss, Thornwood, NY, USA) and mouse retinal sections were examined at 400× by laser confocal microscope (LSCM 510 META; Oberkochen, Germany), maintaining identical settings. We further measured TLR4-immunofluorescence relative intensity in each optical slice along lines around inner layer of blood vessels in human retina samples and transecting inner layer of mouse retina by Image-Pro Plus 6.0 (Media Cybernetics, Silver Spring, Maryland, USA). Image processing was performed with Adobe Photoshop CS 3.0 (Adobe Systems, San Jose, CA, USA).

### Cell culture

HMEC-1 human microvascular endothelial cell line was obtained from American Type Culture Collection (Manassas, VA, USA). HMEC-1 cells were cultured in human endothelial-SFM (5.5 mmol/L glucose, Invitrogen, Carlsbad, CA, USA). Mouse retinal endothelial cells (MRECs) of wild type mice and TLR4 knockout mice were purchased from PriCells (Wuhan, China) and were cultured in endothelial cell complete medium (MED0002 + SUP0002, PriCells) according to the manufacturer’s instructions. Only cells between passages 3 and 8 were used in the study. The glucose was purchased from Sigma-Aldrich, St. Louis, MO, USA. The cells were treated with 5.5 mmol/l normal glucose or with 15 and 25 mmol/l glucose for indicated times in sustained condition. The cells were also pretreated with TLR4 antagonist *Rhodobacter sphaeroides* LPS (LPS-RS) (Invivogen, San Diego, CA, USA) or TLR4 siRNA (Santa Cruz Biotechnology, California, USA) according to the instruction of manufacture followed by 12 h 5.5, 15, or 25 mmol/l glucose treatment. Cell supernatants, lysates and RNA were collected for further experiments.

### RNA extraction and real-time quantitative RT-PCR

HMEC-1 cells or MRECs were cultured in 30-mm tissue culture dishes. After treatment with glucose, total RNA was extracted using Trizol reagent (Invitrogen, Carlsbad, CA, USA) and the cDNA was prepared by reverse transcription according to the manufacturer’s protocol. Real-time PCR was performed on an Applied Biosystems StepOne Real-Time PCR System using the comparative threshold cycle (CT) quantification method. Each reaction contained 12.5 μl of 2× SYBR green Master Mix, 300 nM oligonucleotide primers synthesized by Invitrogen Biotechnology Co. Ltd, (Shanghai, China), 10 μl of 1 in 10 dilution of the cDNA and water, to a total of 25 μl. The thermal cycling conditions included an initial denaturation at 95 °C for 10 min, 40 cycles at 95 °C for 30 s, 60 °C for 1 min, and 72 °C for 1 min. The mRNA expression in each sample was determined after correction with the expression of human GAPDH or mouse GAPDH. Each measurement of a sample was conducted in triplicate. The following primers were used: human GAPDH (70 bp): forward: CCACATCGCTCAGACACCAT; reverse: CCAGGCGCCCAATACG; human TLR4 (138 bp): forward: ATGAAATGAGTTGCAGCAGA; reverse: AGCCATCGTTGTCTCCCTAA; human IL-1β (133 bp): forward: TCCAGGGACAGGATATGGAG; reverse: TCTTTCAACACGCAGGACAG; human VEGF (120 bp): forward: TCCAGGAGTACCCTGATGAG; reverse: ATTCACATTTGTGTGCTGT; human bFGF (184 bp): forward: GAGGAGTTGTGTCTATCAAAG; reverse: GTTCGTTTCAGTGCCACATACC; mouse GAPDH (93 bp): forward: TGAGCAAGAGAGGCCCTATC, reverse: AGGCCCCTCCTGTTATTATG; mouse IL-1β (105 bp): forward: TCCAGGATGAGGACATGAGCAC, reverse: GAACGTCACACACCAGCAGGTTA. All mRNA values were normalized against the levels of human or mouse GAPDH mRNA. The normalized values of treatment groups are expressed as the fold increase over the untreated control cells in y axis.

### Western blotting

HMEC-1 cells treated with glucose were collected and lysed with lysis buffer containing RIPA and phenylmethylsulfonyl fluoride (PMSF). Protein concentration was determined by the Bradford–Lowry method and the samples were stored at −80 °C until used for immunoblot analysis. Protein samples (30 μg) were loaded onto SDS-PAGE gels and then were transferred to a PVDF membrane (Immobilon-P; Millipore, Bedford, MA, USA). After being blocked with 5 % nonfat milk for 1 h, the membrane were incubated with the following primary antibodies at 4 °C overnight: polyclonal rabbit anti-human TLR4 (1:500; Boster Wuhan, China), polyclonal rabbit anti-human MyD88 (1:100; Boster Wuhan, China) and monoclonal mouse anti-β-actin (1:100; Boster, Wuhan, China). Protein bands were visualized by incubation with anti-mouse secondary antibody or anti-rabbit secondary antibody (1:3000; BOSTER, Wuhan, China) and chemiluminescence substrates (ECL Plus; TIANGEN, Beijing, China).

### Flow cytometry

HMEC-1 cells were cultured in 24-well plates for at least 24 h, then were stimulated with 15 mmol/l glucose for another 24 h. The cells were then blocked by 5 % BSA PBS for 30 min, and were incubated with FITC labeled anti-TLR4 antibody (eBioscience Inc., San Diego, CA, USA) for 1 h at 4 °C. After the incubation, cells were scraped and suspended in 500 μl buffer. The intensity of fluorescence was measured using a FACSCalibur and CellQuest software (BD PharMingen).

### Enzyme-linked immunosorbent assays (ELISA)

HMEC1 cells with/without siRNA silence or MRECs of wild type or TLR4 knockout mice were stimulated with glucose. Culture supernatant was harvested, centrifuged to remove cellular debris, and then stored at −80 °C until use. The concentration of IL-1β in the supernatants was measured by human IL-1β ELISA (Boster Corporation, Wuhan, China) or mouse IL-1β ELISA (R&D Systems, Minneapolis, MN, USA) according to manufacturer’s instructions.

### Reproducibility and statistical analysis

All experiments were performed at least three times. Results were highly reproducible. Representative results were shown in figures. Results are expressed as the mean ± SE. Data were analyzed by ANOVA with S–N–K post hoc analyses. All statistical analyses were performed using SPSS20.0 Software. *P* < 0.05 was considered as statistically significant.

## Results

### The expression of TLR4 in premembrane of diabetic retinopathy patients

**Fig. 1:**
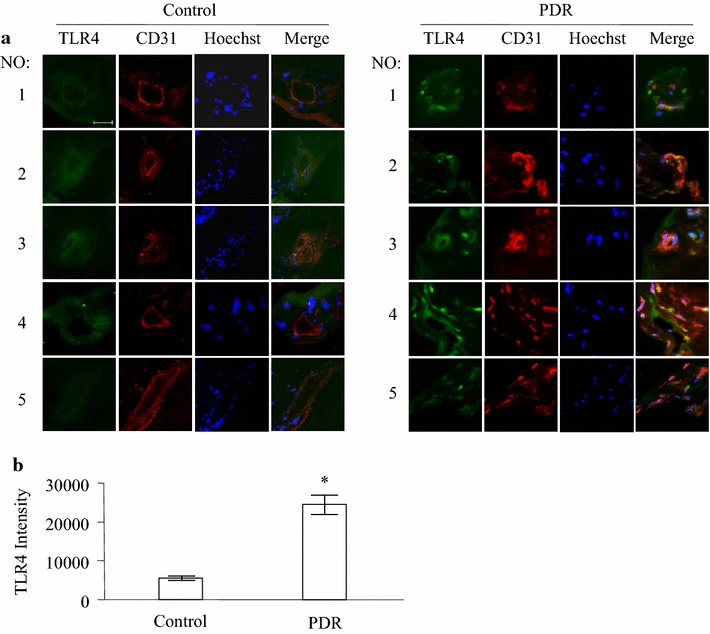
Expression of TLR4 in PDR fibrovascular membrane. Immunofluorescence staining of TLR4 (*green*) and CD31 (*red*) was performed in normal human retinas, fibrovascular membranes from PDR patients. Results show five fibrovascular membranes from patients with PDR and five normal human eye ball controls (**a**). TLR4 immunofluorescence relative intensity in retinal sections was quantified and displayed (**b**). *P < 0.05 compared to the control group. *Scale bar* 20 μm

Our previous study showed that TLR4 played a critical role in inflammation-induced angiogenesis in a mouse model. In this study, we at first examined the expression of TLR4 in the premembranes of patients with diabetic retinopathy. The premembranes of five diabetic retinopathy patients and retinas of five normal human eye balls were examined with immunofluorescence staining. The expression of TLR4 was detected in the retina from normal human eyes (Fig. 1a, Control 1–5) and fibrovascular membranes removed from the eyes of patients with PDR (Fig. 1a, PDR 1–5). Positive staining of CD31, an endothelial marker, was present in both tissues. Colocalization of TLR4 and CD31 was observed in vessel walls. TLR4 expression was significant higher in PDR fibrovascular membranes than in normal retinal vasculatures (Fig. 1b). These data suggest that TLR4 expression is increased in the fibrovasculature and may play a role in the dysfunction of vascular endothelial cells in DR.

### The expression of TLR4 in retinal endothelial cells in STZ-induced diabetic mice

**Fig. 2:**
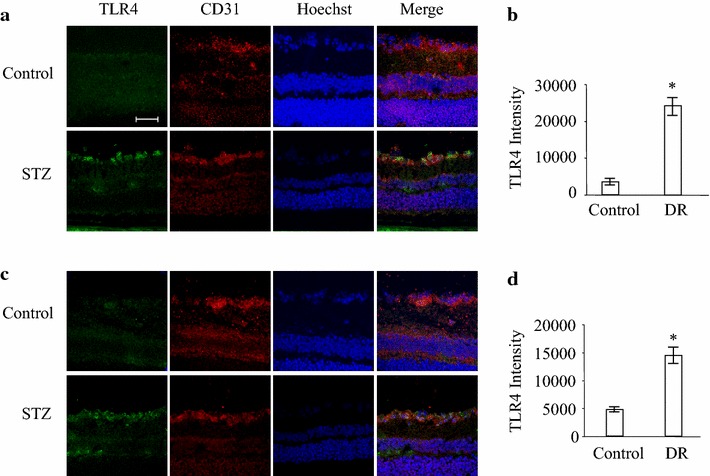
Expression of TLR4 in retinal vessel of diabetic mice. Immunofluorescence staining of TLR4 (*green*) and CD31 (*red*) was performed in normal and diabetic mouse retinas. **a**, **c** Representative results from three independent experiments with four diabetic and control animals in 6 week-group (**a**) or 8 week-group (**c**) after STZ injection are shown. **b**, **d** TLR4 immunofluorescence relative intensity in retinal sections of 6 week-group (**b**) or 8 week-group (**d**) of diabetic mice was quantified. *P < 0.05 compared to the control group. *Scale bar* 50 μm

STZ-induced diabetes in mice is a commonly used model to study nonproliferative DR. We examined the expression of TLR4 in mice 6 and 8 weeks after STZ injection (Fig. 2a, c). Colocalization of TLR4 and CD31 was observed in vessel walls. We found that in the diabetic retina, TLR4 level was significantly increased at 6 and 8 weeks after STZ injection compared to control mice (Fig. 2b, d).

### High glucose culture induced the expression of TLR4 by HMEC-1 cells

**Fig. 3:**
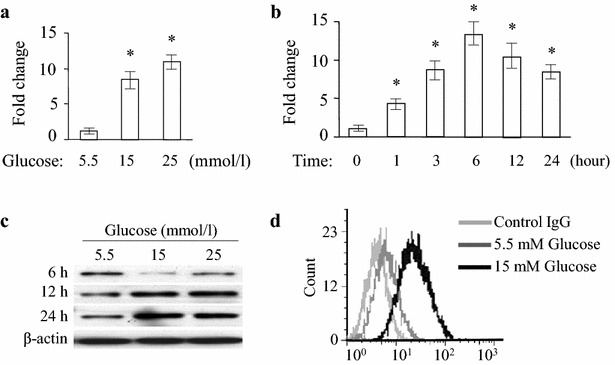
Expression of TLR4 in HMEC-1 cells subjected to high glucose. **a** HMEC-1 cells were stimulated with glucose at the doses of 15 and 25 mmol/l for 6 h. The mRNA for TLR4 was detected by quantitative RT-PCR and normalized to GAPDH. *Asterisk* indicates *P* < 0.05 compared to 5.5 mmol/l glucose. **b** The cells were stimulated with 15 mmol/l glucose for 0, 1, 3, 6, 12 and 24 h. The mRNA of TLR4 was detected by quantitative RT-PCR and normalized to GAPDH and expressed as the mean ± SE. *P < 0.05 compared to the 0 h group. **c** HMEC-1 cells were treated with 5.5, 15 and 25 mmol/l glucose for 6, 12 and 24 h and western blot was performed. β-actin was used as a control. **d** TLR4 expression on HMEC-1 cells challenged with 15 mmol/l glucose for 24 h assessed by flow cytometry

We then used HMEC-1 cells as a model to examine microvascular endothelial cell response to increased concentration of glucose. HMEC-1 cells were exposed to 5.5 (normal), 15 and 25 mmol/l glucose for variable time points. Increased expression of TLR4 mRNA was observed after glucose exposure and the highest level of TLR4 expression was induced by 25 mmol/l glucose (Fig. 3a). The increase in TLR4 expression by HMEC-1 cells in response to glucose was time dependent and reached the maximum at 6 h after treatment (Fig. 3b). Western blotting (Fig. 3c) showed a markedly increase in TLR4 expression at 12 and 24 h after high glucose challenge. Analysis of flow cytometry showed similar effect when glucose was used at 15 and 25 mmol/l (Fig. 3d).

### Induction of MyD88 expression in HMEC-1 cells by high glucose

**Fig. 4:**
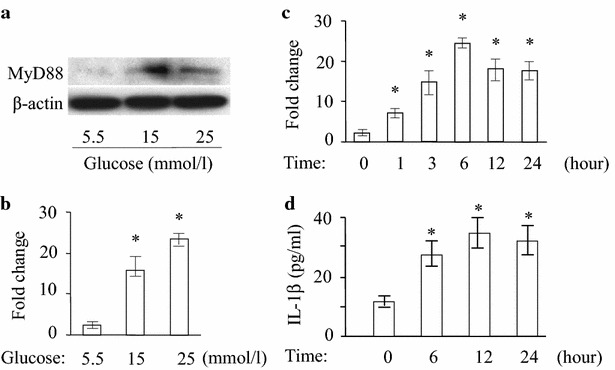
Activation of MyD88 and cytokine expression in HMEC-1 cells under high glucose condition. **a** Western blotting was performed for MyD88 expression in the HMEC-1 cells after exposure to 15 and 25 mmol/l glucose for 12 h. β-actin was used as a control. **b** Cells were treated with 15 and 25 mmol/l glucose for 6 h. The mRNA for IL-1β was detected by quantitative RT-PCR and normalized to GAPDH. *Asterisk* indicates *P* < 0.05 compared to 5.5 mmol/l glucose. **c** The cells were stimulated with 15 mmol/l glucose for 0, 1, 3, 6, 12 and 24 h. The mRNA of IL-1β was detected by quantitative RT-PCR and normalized to GAPDH to the 0 h group and expressed as the mean ± SE. *P < 0.05 compared to the 0 h group. **d** IL-1β protein in the supernatants of HMEC-1 cells after high-glucose treatment for indicated time points measured using ELISA. *Asterisk* indicates *P* < 0.05 compared to 5.5 mmol/l glucose

Activation of MyD88 is a key step in the TLR4 pathway that culminates in NF-κB activation and inflammatory cytokine expression. We examined the effect of high glucose on the expression of MyD88 by HMEC-1 cells using Western blotting. We observed that high glucose increased the expression of MyD88 by HMEC-1 cells at 15 and 25 mmol/l for 12 h (Fig. 4a). These findings indicate that increased TLR4 expression under high glucose exposure induces the expression of MyD88, which may activate inflammatory cascade.

### Increased inflammatory cytokine production by HMEC-1 cells in response to high glucose

To investigate the inflammatory response of TLR4, we determined the expression of inflammatory cytokine IL-1β as well as VEGF and bFGF by HMEC-1 cells under high glucose condition. The level of IL-1β mRNA after 15 and 25 mmol/l glucose treatment was significantly increased compared with treatment with 5.5 mmol/l glucose (Fig. 4b) (*p* < 0.05). Kinetic study showed treatment with 15 mmol/l glucose significantly increased IL-1β mRNA at 3, 6, 12 and 24 h (Fig. 4c). We then measured IL-1β protein in the cultural supernatant of glucose-treated HMEC-1 cells. IL-1β level was significantly increased at 6, 12, 24 h and persisted under high glucose condition (15 mmol/l) (Fig. 4d). Similarly, stimulation of HMEC-1 cells by 15 mmol/l glucose enhanced the expression of VEGF and bFGF (Additional file 1: Figure S1). Therefore, high glucose elevates the secretion of inflammatory and proangiogenic cytokines via activation of TLR4 pathway.

### Inflammatory response activated by high glucose via TLR4 in HMEC-1 cells

**Fig. 5:**
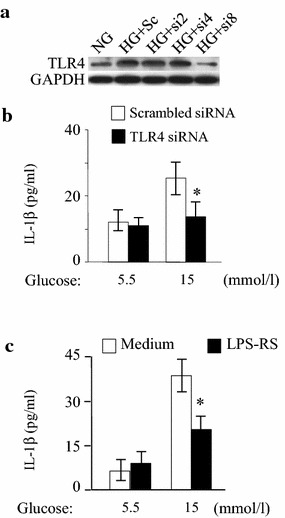
Inhibition of TLR4 reduces high glucose-induced production of IL-1β by HMEC-1 cells. **a** Western blot analysis of TLR4 expression by HMEC-1 cells under high glucose condition after siRNA treatment. *NG* normal glucose, *HG* high glucose, *Sc* scrambled control siRNA, *si2* TLR4 siRNA 0.2 pmol/l, *si4* TLR4 siRNA 0.4 pmol/l, *si8* TLR4 siRNA 0.8 pmol/l. **b** IL-1β concentration in supernatants of HMEC-1 cells after high-glucose treatment in the presence of TLR4 siRNA (0.8 pmol/l) was measured using ELISA. *Asterisk* indicates *P* < 0.05 compared to 5.5 mmol/l glucose. **c** IL-1β level in supernatants of HMEC-1 cells in the presence of TLR4 antagonist LPS-RS (1 μg/ml) after high-glucose treatment was measured using ELISA. *Asterisk* indicates *P* < 0.05 compared to scrambled control RNA

siRNA was used to confirm the role of TLR4 in high glucose-induced inflammatory response. TLR4 expression in HMEC-1 cells was significantly decreased after siRNA transfection (Fig. 5a). This resulted in significant decrease in IL-1β level at 15 mmol/l glucose treatment (Fig. 5b). Scrambled siRNA had no significant effect on IL-1β expression by HMEC-1 cells (Fig. 5b). We also tested the effects of a TLR4 antagonist, *Rhodobacter sphaeroides* LPS (LPS-RS), on high glucose-induced inflammatory response in HMEC-1 cells. LPS-RS reduced the production of IL-1β by HMEC-1 cells at 15 mmol/l glucose treatment (Fig. 5c). These findings confirmed that the effect of high glucose on the production of IL-1β by HMEC-1 cells was TLR4-dependent.

### Reduction of high glucose-induced cytokine production by MRECs from TLR4 deficient mice

**Fig. 6:**
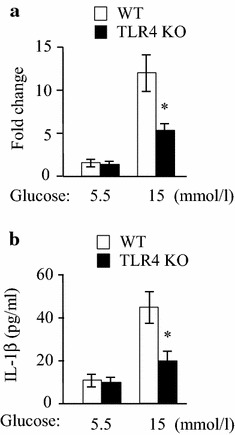
The expression of IL-1β by MRECs from TLR4 deficient mice under high glucose condition. MRECSs of wild type (WT) mice and TLR4 knockout (TLR4 KO) mice were cultured with 5.5 and 15 mmol/l glucose for 6 h (**a**) and 24 h (**b**). **a** The mRNA level of IL-1β was determined by real time PCR. *Asterisk* indicates *P* < 0.05 compared to WT mice. **b** IL-1β protein concentration in the supernatants of MRECs after high-glucose treatment for 24 h was measured using ELISA. *Asterisk* indicates *P* < 0.05 compared to WT mice

Next, we determined the expression of IL-1β under high glucose condition by MRECs from TLR4 deficient mice. Real-time PCR showed that the expression of IL-1β by WT mouse MRECs under 15 mmol/l glucose was increased. However, the expression of IL-1β in MRECs from TLR4 deficient mice was significantly reduced (Fig. 6a) (*p* < 0.05). Similarly, the production of IL-1β protein by MRECs from TLR4^−/−^ mice was also significantly reduced in comparison with WT mouse cells (Fig. 6b) (*p* < 0.05). These data confirm that TLR4 plays a critical role in high glucose-induced inflammatory responses in endothelial cells.

## Discussion

Diabetic retinopathy is a common vascular complication and appears to involve inflammatory responses [30]. Hyperglycemia induces inflammation, affects the production of extracellular matrix and procoagulant proteins, causes apoptosis and inhibits the proliferation of endothelial cells. These events resulted in endothelial dysfunction in diabetic retinopathy [31].

Abnormalities in glucose concentration have been reported to elevate and activate TLR4 to promote the secretion of inflammatory cytokines in mouse mesangial cells and contribute to diabetic nephropathy [14, 23]. The expression and function of TLR4 are elevated in monocytes of diabetic patients [17, 32]. Genetic deficiency of TLR4 is associated with significant reduction of aortic plaque and lower triglyceride accumulation in the heart in early stages of diabetes [13, 33]. In addition, TLR4 gene polymorphism was found to be associated with diabetic retinopathy [15]. Thus, our findings of increased TLR4 in endothelial cells after high glucose exposure provide the evidence for the role of TLR4 in diabetic retinopathy.

Hyperglycemia, particularly the fluctuation of glucose levels, causes a significant oxidative stress, decreasing the expression of endothelial nitric oxide synthase and impairing NO metabolism [34]. Fluctuating hyperglycemia induces an increased production of collagen. Furthermore, fluctuations of glycemia increase endothelial cell apoptosis, endothelial expression of adhesion molecules, and vascular smooth muscle cell proliferation [35]. Thus, glucose fluctuations appear to be more deleterious to vascular cell integrity than constant high glucose concentrations.

Oxidative stress plays a critical role in mediating the upregulation of TLR4 under hyperglycemic conditions. It is reported that hyperglycemia induces TLR4 expression in hyperglycemic human retinal endothelial cells. It also increases both MyD88 and non-MyD88 pathways, nuclear factor-κB (NF-κB), biomediators, and monocyte adhesion. Antioxidant treatment reduced TLR4 expression and downstream inflammatory markers [36]. The articles by Mudaliar et al. [37] and Lu et al. [38] showed that TLR4 play an important role in mediating inflammatory pathways in endothelial cells exposed to high glucose. Therefore, strategies to block TLR4 signaling pathways pose a promising avenue to alleviate diabetic-induced vascular complications.

Hyperglycemia-induced TLR4 expression in human monocytes is associated with increased NAPDH oxidase activity triggered by PKC [17]. High glucose increased PKC-δ activity that mediates a wide range of cellular function including increased translocation of NF-κB [39]. Additionally, PKC-δ stimulates p47Phox, a NADPH oxidase cytosolic subunit, which in turn stimulates the NAPDH oxidase activity to generate ROS critical for TLR4-mediated activation of NF-κB. Furthermore, high glucose was found to enhance the apoptosis of endothelial cells by activation of PKC and NAPDH [17].

## Conclusion

Our study demonstrated that hyperglycemia enhances the expression of TLR4 and activates TLR4 in human endothelial cells that may play an important role in diabetic retinopathy. Thus, targeting TLR4 signaling pathway may be a novel therapeutic approach to vasculature-related disorders.

## Additional file

10.1186/s13098-015-0086-4 HMEC-1 cells were cultured with glucose at the doses of 5.5 and 15 mmol/l for 6 h. The mRNA for VEGF and bFGF was detected by quantitative RT-PCR and normalized to GAPDH. * indicates *P*<0.05 compared to 5.5 mmol/l glucose.

## Abbreviations

DR: diabetic retinopathy
ELISA: enzyme-linked immunosorbent assays
HMEC-1: human microvascular endothelial cells
IL: interleukin
LPS: lipopolysaccharide
MRECs: mouse retinal endothelial cells
LPS-RS: *Rhodobacter sphaeroides* lipopolysaccharide
PAMPs: pathogen-associated molecular patterns
STZ: streptozotocin
TLR4: toll-like receptor 4
