# Supplementary material for: High glucose induces and activates Toll-like receptor 4 in endothelial cells of diabetic retinopathy
Source: Diabetol Metab Syndr. 2015 Oct 13;7:89. doi: 10.1186/s13098-015-0086-4 (PMC4604707; doi:10.1186/s13098-015-0086-4)
Supplement: Supplementary file 1 — 10.1186/s13098-015-0086-4 HMEC-1 cells were cultured with glucose at the doses of 5.5 and 15 mmol/l for 6 h. The mRNA for VEGF and bFGF was detected by quantitative RT-PCR and normalized to GAPDH. * indicates P<0.05 compared to 5.5 mmol/l glucose. [file 13098_2015_86_MOESM1_ESM.ppt]

## Slide 1
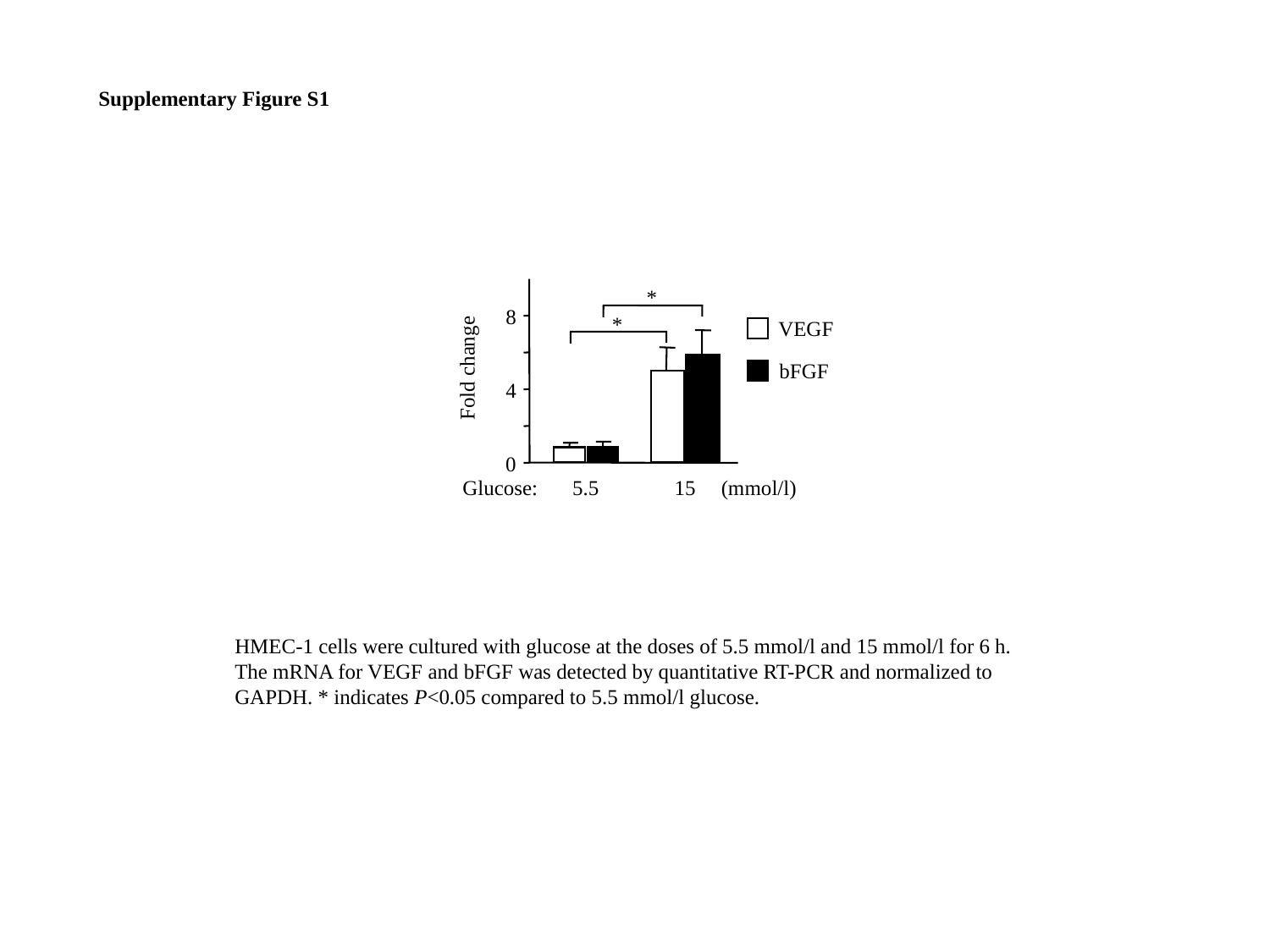

Supplementary Figure S1
*
8
*
VEGF
bFGF
Fold change
4
0
Glucose:
5.5
15
(mmol/l)
HMEC-1 cells were cultured with glucose at the doses of 5.5 mmol/l and 15 mmol/l for 6 h. The mRNA for VEGF and bFGF was detected by quantitative RT-PCR and normalized to GAPDH. * indicates P<0.05 compared to 5.5 mmol/l glucose.
